# On the feasibility of time-resolved X-ray powder diffraction of macromolecules using laser-driven ultrafast X-ray sources

**DOI:** 10.1107/S1600576724005028

**Published:** 2024-07-29

**Authors:** Krishna Prasad Khakurel, Gabriel Žoldák, Borislav Angelov, Jakob Andreasson

**Affiliations:** a Extreme Light Infrastructure ERIC, Za Radnici 835, 25241Dolni Brezany, Czechia; b Center for Interdisciplinary Biosciences, Technology and Innovation Park, P. J. Šafárik University, Trieda SNP 1, 040 11Košice, Slovak Republic; SLAC National Accelerator Laboratory, Menlo Park, USA

**Keywords:** ultrafast X-rays, time-resolved X-ray diffraction, macromolecular structure

## Abstract

With the emergence of ultrafast X-ray sources, interest in following fast processes in small molecules and macromolecules has increased. Most of the current research into ultrafast structural dynamics of macromolecules uses X-ray free-electron lasers. In parallel, small-scale laboratory-based laser-driven ultrafast X-ray sources are emerging. Continuous development of these sources is underway, and as a result many exciting applications are being reported. However, because of their low flux, such sources are not commonly used to study the structural dynamics of macromolecules. This article examines the feasibility of time-resolved powder diffraction of macromolecular microcrystals using a laboratory-scale laser-driven ultrafast X-ray source.

## Abstract 2

This article presents an investigation into the feasibility of X-ray powder diffraction of macromolecules with laser-driven X-ray sources, which may enable in-house sub-picosecond time-resolved structural studies of macromolecules.

## 1. Introduction

Ultrafast science with short-pulse X-ray sources has advanced extensively in recent years. One of the objectives behind the development of such sources is to follow the very short-lived dynamics in material and biological samples (Schoenlein *et al.*, 2019 ▸; Spence *et al.*, 2012 ▸), and to do so with atomic spatial resolution and/or element specificity. X-ray free-electron lasers (XFELs) are used for a major part of this work. However, XFELs are in high demand, and access to them is difficult to secure. Complementary methods to probe the short-lived dynamics in a sample are being developed with the use of small-scale laser-driven ultrafast X-ray sources (Korn *et al.*, 2002 ▸; Albert *et al.*, 2014 ▸; Weisshaupt *et al.*, 2014 ▸; Koç *et al.*, 2021 ▸). Although these sources typically have low flux, they are more easily accessible and can be quickly modified for measurements complementary to the work at XFELs. Furthermore, using pulsed lasers to drive X-rays offers accurate and flexible pump–probe strategies.

While synchrotrons can probe dynamics in macromolecules on the timescale of hundreds of picoseconds (Schulz *et al.*, 2022 ▸; Levantino *et al.*, 2015 ▸), XFELs bring additional value through their ability to explore ultrafast processes with a temporal resolution of a few tens of femtoseconds. Such short-lived dynamics have been probed for both crystalline (Chapman *et al.*, 2011 ▸) and in-solution samples (Arnlund *et al.*, 2014 ▸). Typically, for atomistic details of the ultrafast dynamics in macromolecules, either single-crystal or serial crystal dif­frac­tion is performed, and respective data treatment strategies are adopted to visualize the time-resolved structure of the macromolecules. XFELs have revolutionized the study of bio­molecular dynamics, allowing researchers to investigate pro­cesses such as protein folding, enzyme catalysis, photosynthesis (Spence, 2017 ▸; Li *et al.*, 2024 ▸) and dynamics in other biological systems (Cellini *et al.*, 2024 ▸).

How molecules respond to external stimuli such as light is a fundamental question in biology. The first few processes of such responses occur on very short timescales (Gruhl *et al.*, 2023 ▸). The process of ligand dissociation, breaking of bonds and charge dynamics all occur on sub-picosecond timescales (Barends *et al.*, 2015 ▸; Grånäs *et al.*, 2019 ▸). Furthermore, coherent motion following the excitation by external stimuli occurs on timescales from sub-picoseconds to a few picoseconds, as do proton dynamics in enzymes and quakes in proteins (Yang *et al.*, 2022 ▸; Arnlund *et al.*, 2014 ▸). Probing these dynamics is an active research topic within the XFEL structural biology community. In order to see these changes on an ultrafast timescale, a spatial resolution of 3 Å or better is required (Westenhoff *et al.*, 2022 ▸).

Up to now, no time-resolved crystallography of macromolecules has been demonstrated using laser-driven X-ray sources, although static macromolecule diffraction from such a source was reported by Bonvalet *et al.* (2006 ▸). With source optimization and improvements in laser technology, focusing optics, detectors and sample handling, the efficiency of such an experiment has been improved, and time-resolved macromolecular crystallography of large (>100 µm^3^) high-quality single crystals could be feasible with laser-driven ultrafast X-ray sources (Khakurel *et al.*, 2020 ▸). Experiments to demonstrate this are in progress.

One of the biggest challenges in crystallography is growing high-quality crystals of sufficient size in a reasonable number of trials (Khakurel *et al.*, 2019 ▸). In the early 2000s, a few groups started investigating the possibility of obtaining the structure of macromolecules from X-ray powder diffraction (XRPD) of microcrystals (Von Dreele, 1999 ▸, 2005 ▸, 2007 ▸; Von Dreele *et al.*, 2000 ▸; Margiolaki *et al.*, 2005 ▸) usng both synchrotron (Margiolaki *et al.*, 2005 ▸) and in-house continuous-wave (CW) X-ray sources (Hartmann *et al.*, 2010 ▸). Importantly, new structures of macromolecules have even been reported at a resolution of about 1.5 Å using such methods (Margiolaki *et al.*, 2007*a* ▸), indicating that XRPD can attain a sufficient resolution to be a relevant tool for studying ultrafast dynamics on the sub-ps to tens of ps level.

In a wider perspective, XRPD has been applied as a method for studying virus proteins, particularly when the acquisition of single crystals has proven challenging (Spiliopoulou *et al.*, 2020*a* ▸; Papageorgiou *et al.*, 2010 ▸). This technique provides valuable structural insights into various proteins (Triandafillidis *et al.*, 2023 ▸; Fili *et al.*, 2016 ▸). XRPD has also been employed to study the structural properties of small peptides like octreotide, a pharmacological mimic of natural somato­statin (Fili *et al.*, 2019 ▸). Additionally, XRPD has been used to investigate the influence of physicochemical factors, such as humidity, on protein crystal structures (Trampari *et al.*, 2018 ▸; Logotheti *et al.*, 2019 ▸). Understanding crystal structures is paramount for drug development and manufacturing. In the pharmaceutical industry, where many substances are delivered as powders, XRPD plays a pivotal role in this context, serving multiple purposes such as polymorph screening and offering valuable information about crystalline composition and phases, which impact drug manufacturability, stability and therapeutic efficacy assessments (Spiliopoulou *et al.*, 2021 ▸). Moreover, XRPD is an essential tool in quality control and quality assessment, enabling the monitoring of structural changes and detection of crystalline impurities in pharmaceutical products.

Here, we discuss the feasibility of performing high-resolution time-resolved powder diffraction of microcrystals of macromolecules using laser-driven ultrafast X-ray sources. The development of this technique will open up possibilities for studying ultrafast structural dynamics in macromolecules, complementary to those in place at synchrotrons and XFELs.

## 2. State-of-the-art XRPD of macromolecules

XRPD has been demonstrated with both synchrotron and continuous laboratory X-ray sources. Such experiments have been performed with a parallel beam, with a beam focused on the detector, and with 1D and 2D detectors (Margiolaki *et al.*, 2007*b* ▸). For typical powder diffraction experiments on macromolecules at a synchrotron, the X-ray flux is often of the order of ∼10^12^ photons s^−1^. Using a 1D detector the measurement time required is a few minutes to obtain a high-resolution powder diffraction pattern. With recent developments in 1D detectors, exploiting the capabilities of the MYTHEN at the Swiss Light Source, the acquisition time has been reduced to seconds (Willmott *et al.*, 2013 ▸; Gozzo *et al.*, 2010 ▸). Macromolecular powder diffraction has also been performed with in-house X-ray sources (Hartmann *et al.*, 2010 ▸). They are used in the screening of polymorphs and determination of the unit cell. The data collected from laboratory sources have also been used in structure refinement. The flux of such sources is of the order of 10^8^ photons s^−1^. Radiation damage is always an issue while collecting diffraction patterns from protein samples. In order to decrease radiation damage on the sample, high-resolution 2D detectors have been used to minimize the data collection time. Furthermore, the sample has been spun during the experiment to reduce the radiation damage at a single point by continuously exposing a fresh part of the sample. Representative numbers for the photon flux and data collection times for XRPD measurements of macromolecules are shown in Table 1 ▸.

## 3. State-of-the-art laser-driven X-ray sources

**Figure 1:**
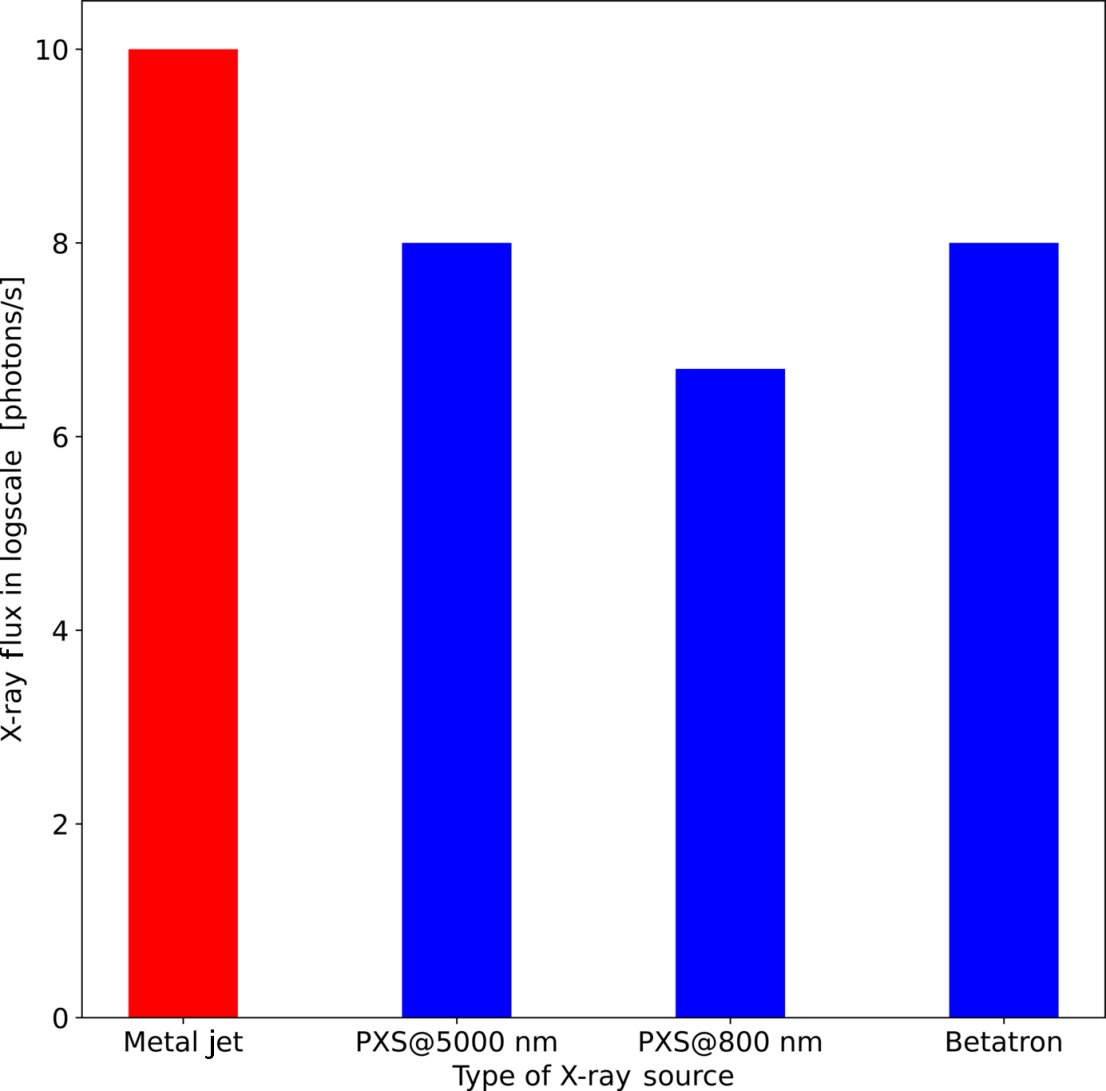
A comparison of the flux of state-of-the-art continuous and pulsed (laser-driven) laboratory X-ray sources. The red color indicates the continuous source and the blue color the laser-driven pulsed source.

Laser-driven X-ray sources typically deliver 10^6^–10^8^ photons s^−1^. A state-of-the-art laser-driven *K*α source delivers about 10^8^ photons s^−1^ (Koç *et al.*, 2021 ▸). The laser technology used to drive the source reported by Koç *et al.* (2021 ▸) is still in a relatively early stage of development, and improvements in both repetition rate and pulse energy can be expected. Furthermore, the X-ray flux reported by Koç *et al.* (2021 ▸) is obtained from a solid target (Cu tape). Considering the increase in flux that has been achieved by switching to a liquid metal jet target in continuous sources (Fig. 1 ▸), it can be expected that a future laser-driven source with a liquid metal jet target driven by a mature 5 µm laser will produce a considerable further increase in X-ray flux.

Other variants of laser-driven ultrafast X-ray sources, such as betatron X-rays, provide ∼10^7^ photons s^−1^ after monochromatization (Chaulagain *et al.*, 2022 ▸). However, the added benefit they provide for time-resolved X-ray measurements is the ultrashort pulse duration [<10 fs in contrast to a few hundred femtoseconds for the solid target/metal jet based plasma X-ray sources (PXS)]. The broad, synchrotron-like spectrum of laser-driven betatron sources also makes them highly suitable for spectroscopy applications. In addition, low-flux ultrafast X-ray sources are available at a small number of synchrotron facilities, for instance, Femtomax at the Max IV synchrotron (Enquist *et al.*, 2018 ▸).

## 4. X-ray powder diffraction with laser-driven X-ray sources: feasibility and calculations

XRPD at synchrotrons is performed with a parallel beam, with focused X-rays, and with both 1D and 2D detectors. With a photon flux of 10^12^ photons s^−1^, a measurement time of 2–3 min is required in order to get a high-resolution powder diffraction pattern. Using 2D detectors the measurement time can be substantially reduced. XRPD from protein microcrystals with a rotating-anode source has been demonstrated to provide high-resolution diffraction patterns in an acquisition time of a few hours (Frankær *et al.*, 2011 ▸). Taking such work as a reference, high-resolution XRPD with a laser-driven X-ray source appears feasible.

From the semi-empirical formula given by Holton & Frankel (2010 ▸), the integrated photons per spot intensity at a particular resolution is given by
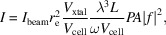
where *I* is the integrated spot intensity (photons per spot), 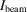 is the intensity of the incident beam (photons s^−1^ m^−2^), 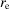 is the classical electron radius (2.818 × 10^−15^ m), 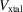 is the illuminated volume of the crystal (in m^3^), 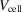 is the volume of the crystal unit cell (in m^3^), λ is the X-ray wavelength (in m), ω is the angular velocity of the crystal (radians s^−1^), *L* is the Lorentz factor (speed/speed), *P* is the polarization factor (photons/photons), *A* is the X-ray transmittance of the path through the crystal to the spot (photons/photons) and *f* is the structure factor of the unit cell at the reciprocal-lattice point of interest (electron equivalents).

**Figure 2:**
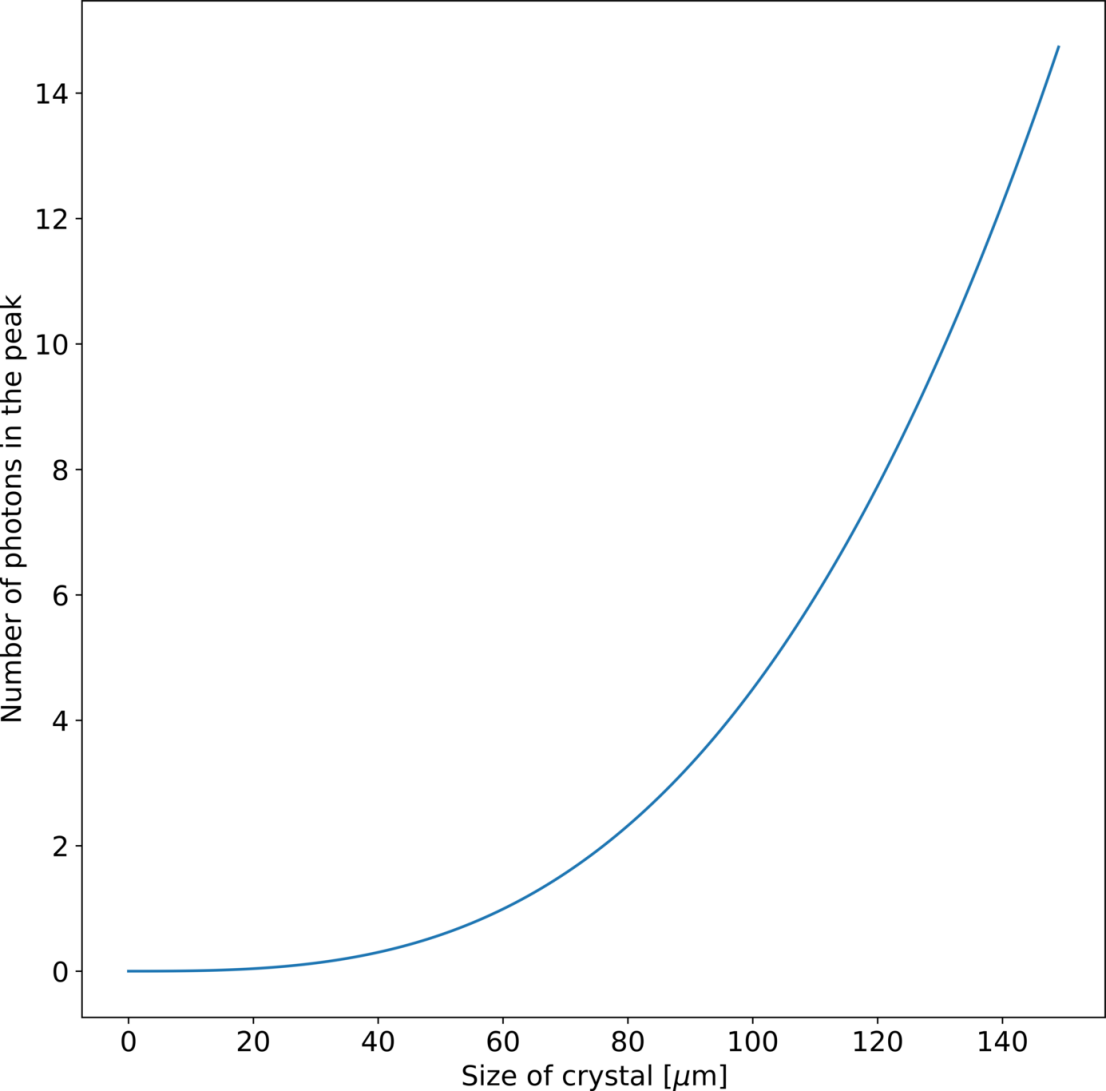
A plot of the expected crystal size required versus the number of photons per peak at 2 Å resolution assuming an X-ray source of flux 10^8^ photons s^−1^.

Considering an average flux of 10^8^ photons s^−1^ at 1.54 Å, a spherical crystal of 50 Å unit-cell length in each dimension and an expected number of photons per spot at 2 Å of 4, then a crystal of radius ∼98 µm is required (Fig. 2 ▸). Here we assume the average empirical formula for a protein H_49.8_C_31.8_N_8.56_O_9.54_S_0.249_ for the calculation of the form factor and absorption.

## 5. Sample consumption in protein crystallography: a comparison across different techniques

One of the concerns in protein crystallography is that the volume of sample needed for the experiment can be large if the sample consumption is high. In a typical single-crystal X-ray diffraction experiment, a few hundred micrograms of proteins are enough to solve the structure. In serial-crystallography-based time-resolved X-ray structural studies of proteins, a few milligrams of the sample are required to obtain the dynamical structure of the proteins. The amount of sample can be reduced if fixed targets are used in delivering the sample to the X-ray beam (Norton-Baker *et al.*, 2021 ▸). For XRPD of macromolecules, a few milligrams of sample or about 10 µl of precipitate is required to obtain the structure (Frankær *et al.*, 2011 ▸; Spiliopoulou *et al.*, 2020*b* ▸). For time-resolved structural studies on a powder sample using laser-driven sources, the available information on sample consumption is still scarce. If we assume that it is necessary to measure a few time points to begin mapping out dynamical processes, then <1 mg of the sample can be sufficient. However, the exact amount of the sample depends on the specific nature of the protein under study and also the number of time points needed to characterize the relevant transient(s). Hence, the sample consumption is certainly larger than in the case of typical single-crystal diffraction but is comparable to XFEL-based serial crystallography.

## 6. Design and setup for time-resolved X-ray powder diffraction with laser-driven X-ray sources

**Figure 3:**
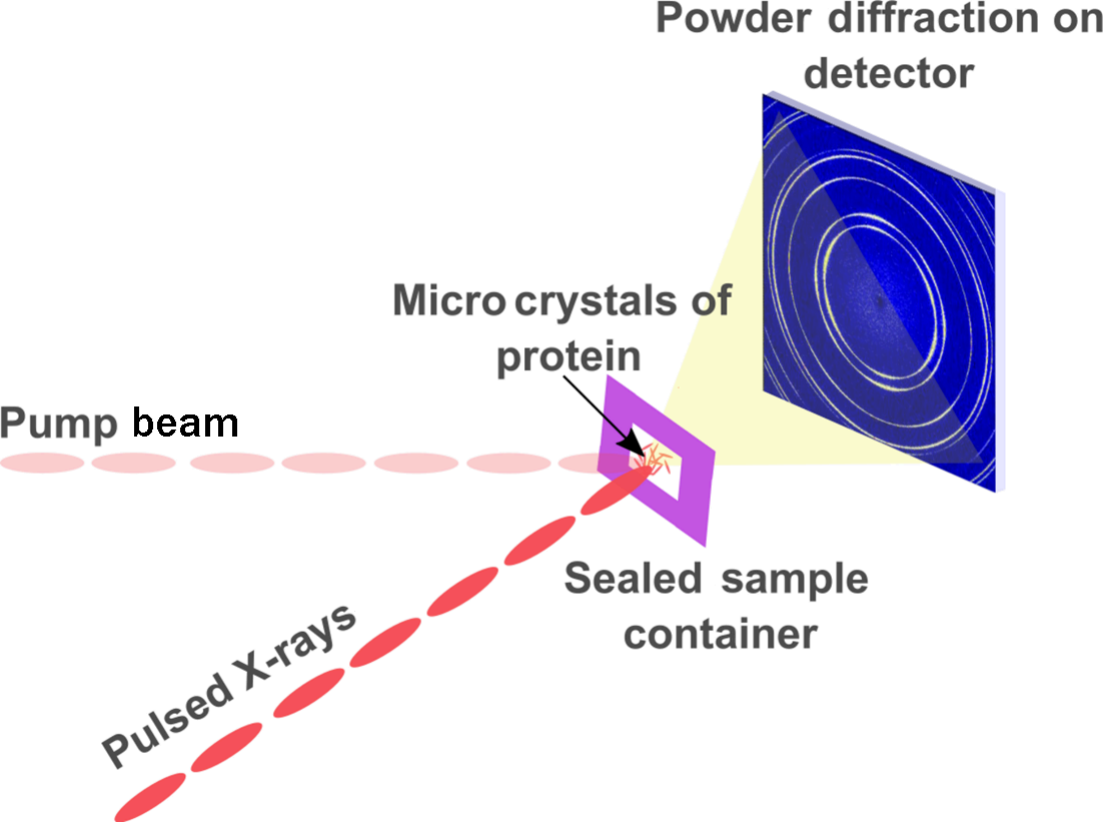
A schematic view of an experimental setup for time-resolved powder diffraction experiments.

A schematic representation of a time-resolved XRPD experiment with a laser-driven X-ray source is shown in Fig. 3 ▸. An ultrashort laser source is used to generate the X-rays. A fraction of the laser pulse energy (typically hundreds of µJ) is split off from the main pulse and is used to pump the sample. A suitable delay line introduces the delay between the pump and the probe beam. Using one laser in a conventional split-and-delay setup, a timing jitter between pump and probe pulses of a few fs can be routinely achieved. Alternatively, by using two laser amplifiers seeded by the same oscillator, time delays up to a full ms between amplifier pulses can be achieved, still with fs precision, as has been verified in optical spectroscopy measurements (Liu *et al.*, 2023 ▸).

The X-rays are spectrally filtered and quasi-collimated using an X-ray multilayer mirror. The multilayer mirror should be carefully designed to provide the optimal conditions for X-ray data collection. The sample can be placed at the focus of the X-ray optics or, in order to optimize the resolution and signal-to-noise ratio, the geometry of the experiment can be set up such that the focus of the X-ray optics can be made exactly on the detector. The sample needs to be mounted on a spinning disc in order to provide a fresh sample for each time delay. The design of a sample holder made from a low-*Z* material such as graphene will contribute to improving the signal-to-noise ratio. Further, approaches such as plunge freezing for small microcrystals can improve the signal-to-noise ratio by reducing the background from the excess solution around the sample (Crawshaw *et al.*, 2021 ▸). In order to increase the number of peaks required for the indexing and refinement, the sample needs to be mounted on a rotation stage and tilted roughly ±30°. The optimal choice of the tilt angle depends on the experiment and also on factors such as the symmetry and shape of the crystal. For instance, crystals having preferred orientation demand a greater tilt angle than those with random orientation. A large-area 2D detector, such as an Eiger or Jungfrau, can be used to collect the powder diffraction. Ensuring either a humidified helium or a vacuum environment between the sample and detector can reduce the absorption of the scattered X-rays, hence improving the efficiency of the measurement.

An interesting alternative to optical excitation of the sample is to use a high-power THz field. Early experiments at 120 Hz have been carried out at the LCLS XFEL where a laser with a pulse enegy of 20 mJ was used to generate THz pulses with peak field up to ∼880 kV cm^−1^ in an experiment on phonon up-conversion in LiNbO_3_ (Kozina *et al.*, 2019 ▸). With the availability of optical kHz drive lasers with several tens of mJ pulse energy, these kinds of experiments are feasible at laser-driven sources at 1 kHz repetition rate. The development of this technology can also find use in the study of THz activated macromolecular dynamics (Lundholm *et al.*, 2015 ▸). A general benefit of using microcrystals in time-resolved X-ray studies is that it ensures a more homogeneous excitation of the samples and, therefore, more uniform occupancy of the excited state under study.

## 7. Limitations related to the stability of laser-driven X-ray sources

A challenging limitation of the proposed method lies in the longer acquisition times, which can be several hours or even days, and thus the requirement for high stability of the laser-driven X-ray sources. One emerging way to address the stability of the source is through the employment of artificial intelligence (AI). AI-assisted laser wakefield-based X-ray/electron sources are under development and have been demonstrated to be operational in a stable manner for several days (Shalloo *et al.*, 2020 ▸). Such technologies can be transferred to other variants of laser-driven X-ray sources. For instance, the environmental parameters influencing the laser pointing instability can be predicted using properly trained machine learning models. These can be used in the self-correction of any instability in the driving laser which can lead to improvement in the stability of the X-ray source. Further, the X-ray source from the laser plasma interaction is largely dependent on the laser intensity, the pulse shape and the spectral component which is constantly evolving in the experiment. With properly designed models these parameters can be optimized for a stable configuration. The data necessary for training the model can be generated from high-repetition-rate lasers.

High-repetition-rate lasers are under rapid development. The parameters above are summarized for presently available kHz lasers. Moderate-power (pulse energies up to a few mJ) laser systems with a repetition rate of up to 100 kHz have already been demonstrated (Hädrich *et al.*, 2022 ▸); these can increase the flux and thus reduce the measurement times correspondingly.

## 8. Conclusion

In conclusion, we have investigated the feasibility of using compact short-pulse laser-driven X-ray sources for time-resolved XRPD of microcrystals of macromolecules. We show that, within certain limitations, powder diffraction of macromolecular crystals to high resolution with such sources is feasible and may open up a range of possibilities for exploring sub-picosecond dynamics in macromolecules. With longer collection times and the optimization of the sources and instruments discussed in the preceding sections, macromolecular structures at ∼3 Å or better resolution can be obtained with the laser-driven XRPD of macromolecules; this will enable the probing of dynamics in macromolecules at the timescales of picoseconds and sub-picoseconds (Westenhoff *et al.*, 2022 ▸). The development of this capability will complement current efforts at making ultrafast molecular movies of macromolecular dynamics, currently ongoing at XFELs. However, the proposed method is strictly aimed at laser-driven X-ray sources and may not be the ideal method where either a single-crystal or serial crystal approach can solve the structure of macromolecules from microcrystals. The method may also open up time-resolved macromolecular crystallography with MeV ultrafast electron sources (Weathersby *et al.*, 2015 ▸; Clabbers *et al.*, 2022 ▸; Fitzpatrick *et al.*, 2013 ▸; Miller, 2014 ▸), where the space-charge effect may present challenges in obtaining a pulsed electron beam of fluence necessary for a single-crystal or serial crystal diffraction experiment. However the requirements pertaining to sample sizes, particularly thickness, may differ significantly between samples.

## Figures and Tables

**Table 1:** A representative summary of the parameters used in X-ray powder diffraction of macromolecules where the structures have been solved

Flux (photons s^−1^)	Collection time (min)	Resolution (Å)	Reference
3 × 10^12^	2	2.27	Margiolaki *et al.* (2007*a* ▸,*b* ▸)
3 × 10^12^	1	2.7	Margiolaki *et al.* (2013 ▸)
1 × 10^8^	∼240	∼4	Frankær *et al.* (2011 ▸)
